# Actinomycosis of the Tongue Mimicking a Benign Tumor: A Case Report With Ultrasonographic and Histopathological Findings

**DOI:** 10.1155/crid/8093513

**Published:** 2026-07-22

**Authors:** Yudai Shimojukkoku, Miki Katsurano, Jun Sumino, Kunihiro Yoshida, Hiroki Masuda, Hiroaki Kanda, Shigeo Tanaka, Ayataka Ishikawa, Kazuhiro Yagihara

**Affiliations:** ^1^ Department of Oral Surgery, Saitama Cancer Center, Saitama, Japan; ^2^ Department of Pathology, Saitama Cancer Center, Saitama, Japan

**Keywords:** actinomycosis, oral cavity, sulfur granules, tongue, ultrasonography

## Abstract

We report a rare case of actinomycosis arising in the tongue. A female patient in her 70s presented to our department with swelling of the tongue. Despite only minimal mucosal change, a submucosal indurated mass was observed. Contrast‐enhanced magnetic resonance imaging revealed a well‐circumscribed mass‐like lesion within the tongue muscle. Ultrasonography showed punctate hyperechoic foci within a hypoechoic lesion. As there were few signs of infection, a benign tumorous lesion was suspected, and an excisional biopsy was performed. Histopathological examination indicated sulfur granules in the mass, and no neoplastic lesion was observed. The mass was ultimately diagnosed as tongue actinomycosis. Three years after surgery, there was no evidence of recurrence at the wound site. This case highlights the diagnostic difficulty of lingual actinomycosis and suggests that internal punctate hyperechoic foci on ultrasonography may provide a useful clue to the diagnosis.

## 1. Introduction

Actinomycosis is a chronic granulomatous infection caused by Actinomyces species, among which *Actinomyces israelii* is one of the best known causative organisms [1]. As these species colonize the oral cavity, the head and neck are the most common regions; approximately 50% of cases occur in this area [2]. Infection typically involves the jaw, parotid region, and cervical soft tissues, whereas cases arising within the tongue musculature are rare [3, 4].

Improvements in public health conditions in many countries have resulted in a decrease in the incidence of actinomycosis [5]. Nevertheless, oral actinomycosis remains diagnostically challenging because it may present as localized swelling with minimal inflammatory change and can therefore mimic a benign or malignant tumor [6, 7]. We report a rare case of actinomycosis arising in the tongue musculature that was initially suspected to be a benign tumor.

## 2. Case Report

A 70‐year‐old woman experienced discomfort in the right posterior tongue starting in November 2021. Because the symptoms persisted for 6 months without improvement, she consulted her primary care physician and was referred to our hospital.

**Figure 1:**
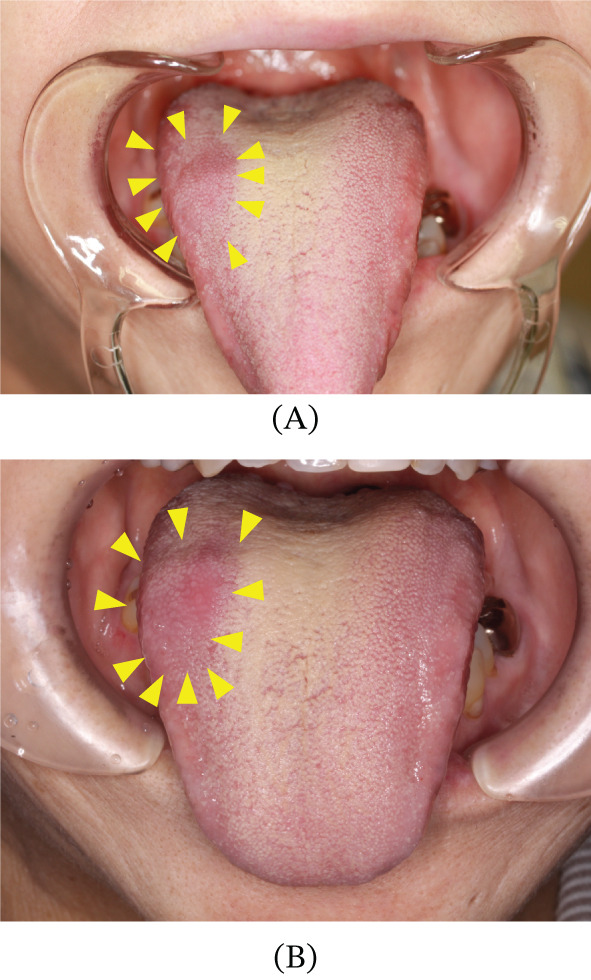
Oral findings. (A) Slight erythematous change with minimal elevation was observed on the right posterior dorsum of the tongue. The remaining dorsal tongue mucosa showed no abnormal findings. (B) Intraoral image obtained during surgery. The lesion showed slight enlargement without obvious change in the mucosal surface.

She had a history of treated hypertension and lumbar spondylolisthesis. She had no history of malignant disease, benign tumors, or trauma. She was a nonsmoker, consumed alcohol approximately once a week, and maintained good oral hygiene. Regarding possible dental and local predisposing factors, there were no obvious clinical findings suggestive of active periodontal infection, such as gingival redness or swelling. Panoramic radiography showed no marked alveolar bone resorption or periapical radiolucency. The patient did not use dentures, and no obvious scar tissue suggestive of repeated tongue biting was observed. Therefore, no clear dental infectious focus or local traumatic factor was identified on clinical examination. At her first visit, there was no facial asymmetry or cervical lymphadenopathy. However, a 10 × 10 mm round, elastic‐hard mass was observed on the right posterior dorsum of the tongue (Figure 1A). The mass was not adherent to the surrounding tissues. The surface was slightly red, but no ulcer was present.

**Figure 2:**
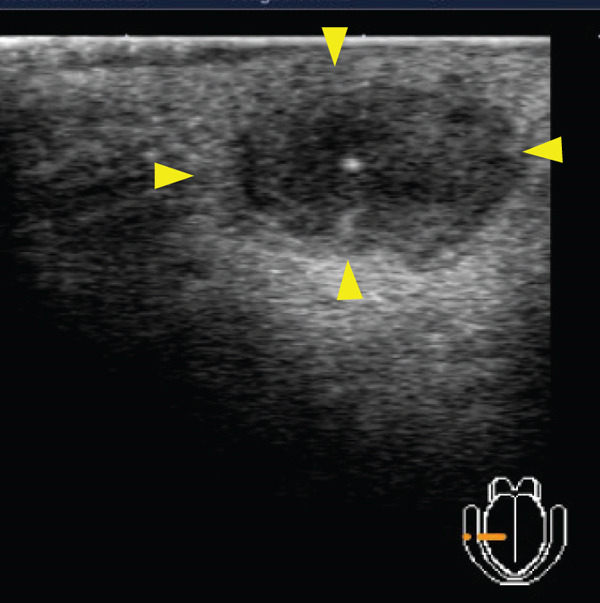
Ultrasonographic image at first visit. The image revealed a punctate hyperechoic focus within a hypoechoic lesion.

**Figure 3:**
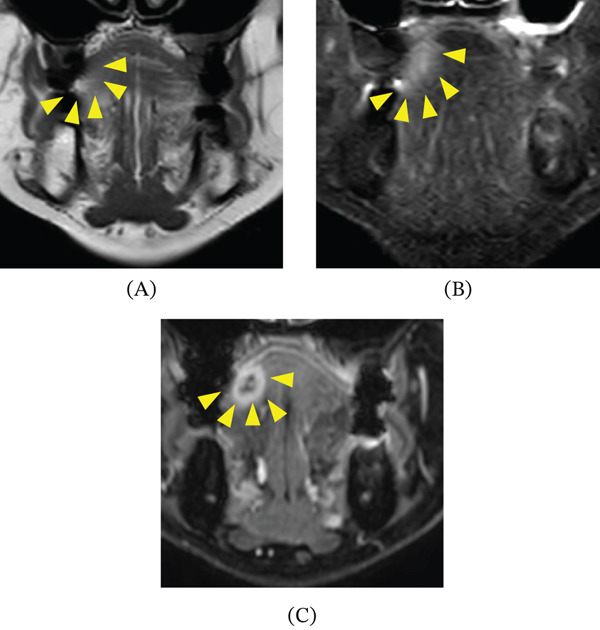
MRI findings. (A) T1‐weighted image showing low signal intensity. (B) STIR image showing faint high signal intensity. (C) Contrast‐enhanced T1‐weighted image showing peripheral enhancement with relatively low central enhancement.

Ultrasonography demonstrated a well‐defined, approximately 11 × 11 mm hypoechoic lesion in the submucosa of the right side of the tongue, containing punctate internal hyperechoic foci (Figure 2). Contrast‐enhanced magnetic resonance imaging (MRI) showed low signal intensity on T1‐weighted images (Figure 3A) and an 11‐mm spherical area of faint high signal intensity in the submucosal layer of the tongue on short tau inversion recovery (STIR) sequences (Figure 3B). Gadolinium‐enhanced T1‐weighted images demonstrated peripheral enhancement of the lesion, whereas the central portion showed little enhancement (Figure 3C). Significant lymph node enlargement was not observed in the neck.

Blood tests indicated slightly elevated C‐reactive protein (CRP) and white blood cell (WBC) levels, suggesting inflammation: CRP was 0.32 mg/dL, and WBC was 8980/*μ*L. Her HbA1c level exceeded 7.0%, which was above the diagnostic threshold of 6.5% for diabetes mellitus. All other laboratory findings were within normal limits.

**Figure 4:**
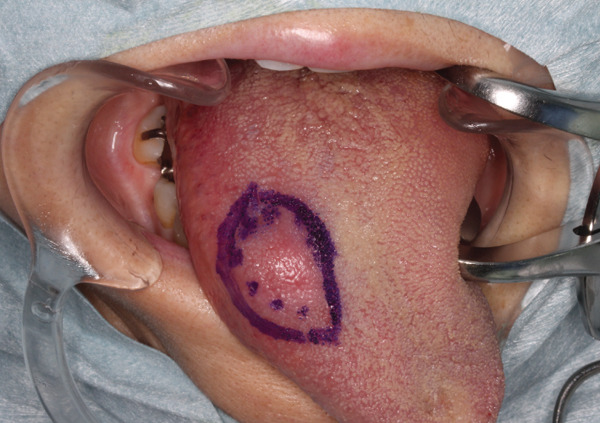
Intraoperative findings. The dotted line delineates the margin of the submucosal lesion, and the solid line indicates the extent of resection.

We initially suspected a benign tumor of the tongue, such as a fibroma or neurilemoma, based on the clinical features and imaging results. Because the mass was relatively small, we decided to perform excisional biopsy under general anesthesia. At operation in July 2022, the size of the lesion had increased slightly since the initial examination (Figure 1B). Intraoperative ultrasonography showed no substantial change in its internal architecture. Because the overlying mucosa was adherent to the lesion, the mass was excised with an approximately 3‐mm margin (Figure 4). No abnormal findings were observed in the deep muscle layer at the resection site. Cefazolin was administered for 24 h postoperatively as standard perioperative prophylaxis, and no additional antibiotic therapy was given thereafter.

**Figure 5:**
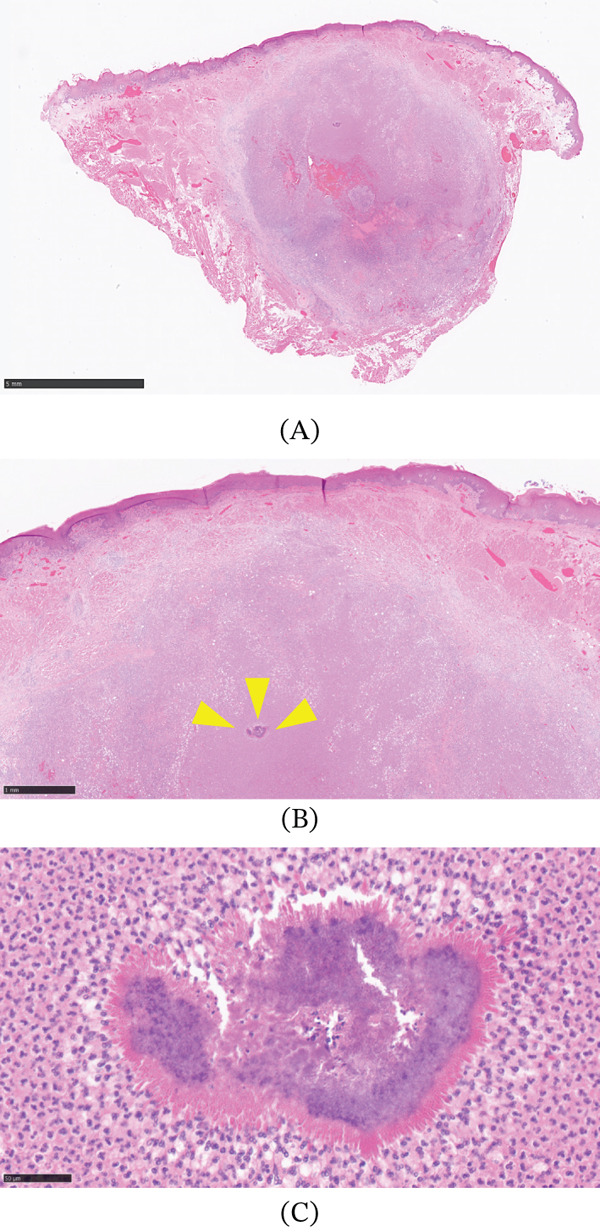
Histopathological examination of the excised specimen stained with hematoxylin and eosin. (A) Low‐power overview; scale bar = 5 mm. (B) Small bacterial aggregates within the granulation tissue were separated from the overlying epithelium; scale bar = 1 mm. (C) Actinomycotic colony; scale bar = 50 *μ*m.

Low‐power histopathological examination revealed a 10 × 10 mm area of granulation tissue with abscess formation in the submucosa. Histopathological findings suggestive of prior trauma or chronic mechanical irritation, such as prominent fibrotic changes, were not identified. Many macrophages, lymphocytes, plasma cells, and multinucleated giant cells were observed around the granulation tissue. A few sulfur granules were observed inside the abscess. No acid‐fast bacilli or fungal organisms were identified (Figure 5A–C), and there was no malignancy in the specimen. Based on these findings, the pathological diagnosis was actinomycosis.

The postoperative course was uneventful. Three years after surgical excision, there was no evidence of recurrence, and she was satisfied with the outcome.

## 3. Discussion

Actinomycosis is caused by Actinomyces species, which are gram‐positive anaerobic bacteria that normally colonize the human oral cavity. Oral actinomycosis is a rare chronic suppurative granulomatous infection, most commonly associated with *A. israelii* and *Actinomyces gerencseriae* [1, 7]. Actinomycosis most commonly involves the cervicofacial region, followed by the thoracic and abdominopelvic regions [7]. Although oral actinomycosis has occasionally been reported, involvement of the tongue is particularly rare. Since 2000, only 17 cases of tongue actinomycosis, including the present case, have been reported worldwide (Table 1) [3, 8–20]. Among these, excision was performed in 10 cases, and most patients were treated with antibiotics such as penicillin, minocycline, or tetracycline. The outcomes were generally favorable, with no recurrence or relapse, likely reflecting the effectiveness of appropriate antimicrobial therapy.

**Table 1:** Reported cases of tongue actinomycosis in the literature, including the present case.

Cases	Years	Author	Patients age	Sex	Clinical diagnosis	History of trauma	Immune deficiency	Use of antibiotics	Treatment
1	2000	F.J. Alamillos‐Granados et al. [8]	74	Female	Malignant tumor	No	DM	No operation	Medicine
2	2006	Atespare et al. [9]	52	Female	Benign/malignant tumor	No	No	Amoxicillin, clavulanic acid	Medicine, excision
3	2006	Enoz M et al. [10]	39	Female	Benign/malignant tumor	No	No	No operation	Medicine
4	2008	Habibi et al. [11]	54	Female	Benign tumor	Yes	No	Penicillin	Medicine, excision
5	2011	Kurtaran et al. [12]	54	Female	Tumor	Yes	Not described	Amoxicillin, clavulanic acid	Medicine, excision
6	2013	Escoda et al. [13]	49	Male	No described	No (but erosion with richen)	No	No operation	Medicine
7	2017	Rocha et al. [14]	44	Female	Vascular malformation	Yes	No	Not described	Debridement
8	2017	Jat et al. [15]	44	Female	Malignant tumor	No	No	Doxycycline	Medicine, excision
9	2017	Aneja et al. [16]	14	Male	Malignant tumor	Yes	No	Amoxicillin, clavulanic acid	Excision
10	2018	Sadeghi et al. [17]	66	Male	Chronic inflammatory diseases	No	No	No operation	Medicine
11	2018	Bekerecioglu et al. [18]	45	Female	Benign tumor	No	No	Penicillin	Medicine, excision
12	2019	Siddiq et al. [19]	60	Female	Malignant tumor	No	No	No operation	Medicine
13	2020	D′Amore F et al. [20]	52	Male	Papilloma	No	No	Clarithromycin	Medicine, excision
14	2020	Al‐Rawee RY et al. [3]	65	Female	Benign tumor	No	No	Amoxicillin, clavulanic acid	Excision
15	2021	Farquharson A [21]	64	Female	Leukoplakia	Yes	No	No operation	Laser/medicine
16	2023	Reyes MRT et al. [22]	56	Female	Nodular median rhomboid glossitis	No	No	Amoxicillin	Excision
17	2025	Author	70	Female	Benign tumor	No	DM	Cephazolin	Excision

*Note:* Clinical diagnosis indicates the provisional diagnosis at presentation. The present case is included in this summary.

Abbreviations: DM, diabetes mellitus; NR, not reported.

The antibiotic management in the present case should be interpreted in the context of the clinical course. Because the lesion showed few clinical signs of infection and was clinically and radiologically suspected to be a benign tumor of the tongue, actinomycosis was not included in the preoperative differential diagnosis. Excisional biopsy was therefore performed for diagnostic and therapeutic purposes. Perioperative antimicrobial prophylaxis was administered according to our institutional protocol; cefazolin was given for 24 h postoperatively, which is consistent with general surgical antimicrobial prophylaxis guidelines recommending discontinuation of prophylactic antibiotics within 24 h after surgery [23]. Until the histopathological diagnosis became available, the patient was managed as having a suspected benign tongue tumor.

Histopathological examination subsequently revealed sulfur granules within an abscess, leading to the diagnosis of actinomycosis. Although prolonged penicillin‐based antibiotic therapy is commonly recommended for actinomycosis, the lesion had already been completely excised, no abnormal findings were observed in the deep muscle layer at the resection site, and there was no clinical evidence of recurrence or infectious relapse during follow‐up. Therefore, additional prolonged antibiotic therapy was not administered. This clinical course should not be interpreted as evidence that prolonged antibiotic therapy can be routinely omitted in tongue actinomycosis. Rather, it indicates that, in this selected case, the lesion was clinically controlled after complete surgical excision with careful long‐term follow‐up.

The clinical and imaging findings in this case illustrate the diagnostic difficulty of tongue actinomycosis. Histopathological examination revealed a small number of sulfur granules within the granulation tissue, leading to a diagnosis of actinomycosis rather than a benign tumor. Previous reports have noted that tongue actinomycosis is difficult to distinguish from benign tumors because of its nonspecific clinical presentation [1, 3, 20]. In our case, the patient presented with swelling of the tongue and slight redness of the surface, but there was no ulceration or pain. MRI and ultrasonography revealed a well‐defined round mass without clear invasion of the surrounding tissue. As these findings may also be seen in benign soft tissue tumors, it was difficult to differentiate actinomycosis from a benign tumor. However, ultrasonography also demonstrated punctate internal hyperechoic foci, which may reflect sulfur granules and thus serve as a possible clue to the diagnosis of actinomycosis.

Kolm et al. and Vazquez et al. reported cases of actinomycosis in patients with human immunodeficiency virus (HIV) infection [24, 25]. Although Actinomyces species are commensal organisms of the oral cavity with relatively low virulence, impaired host immunity may increase susceptibility to infection. In the present case, the elevated HbA1c level suggested previously unrecognized diabetes mellitus, which may have contributed to the development of actinomycosis. However, because only a limited number of cases of lingual actinomycosis have been reported, the association between impaired host immunity and this condition remains unclear. Further accumulation of cases is therefore needed to clarify whether immunocompromised status is a predisposing factor for actinomycosis of the tongue. Some recent reports have suggested that Actinomyces species may occasionally disseminate hematogenously and establish secondary foci in distant organs [26–28]. Nevertheless, actinomycosis is generally considered to arise through contiguous spread after disruption of the mucosal barrier. In the present case, although hematogenous spread from an occult dental source cannot be completely excluded, direct invasion through minor mucosal injury appears to be a more plausible explanation.

This case report has several limitations. First, microbiological culture was not performed; therefore, species‐level identification of Actinomyces was not available. Although the diagnosis was supported by the histopathological finding of sulfur granules within the abscess and by the absence of malignant, acid‐fast bacterial, or fungal findings, the lack of culture prevented species‐level microbiological confirmation of the causative organism. Second, although no obvious dental infectious focus, denture‐related irritation, repeated bite trauma, or histopathological evidence of chronic mechanical irritation was identified, subtle mucosal injury or alterations in the oral microbiota could not be completely excluded because microbiological assessment of the oral flora was not performed.

Lingual actinomycosis should be included in the differential diagnosis of a well‐defined submucosal tongue mass, particularly when ultrasonography reveals punctate internal hyperechoic foci. Histopathological examination remains essential for definitive diagnosis.

## Funding

No funding was received for this manuscript.

## Ethics Statement

This study was conducted in accordance with the Declaration of Helsinki and was approved by the institutional ethics committee of our hospital (Approval No. 1597).

## Consent

Written informed consent was obtained from the patient for publication of this case report and the accompanying images.

## Conflicts of Interest

The authors declare no conflicts of interest.

## Data Availability

The data supporting the findings of this case report are available from the corresponding author upon reasonable request.

## References

[1] Shigeoka M. , Takeda D. , and Akashi M. , Actinomycosis of the Lower Lip: Report of a Case, Case Reports in Dentistry. (2022) 2022, no. 1, 6121315, 10.1155/2022/6121315, 35957626. 35957626 PMC9363213

[2] Karanfilian K. M. , Valentin M. N. , Kapila R. , Bhate C. , Fatahzadeh M. , Micali G. , and Schwartz R. A. , Cervicofacial Actinomycosis, International Journal of Dermatology. (2020) 59, no. 10, 1185–1190, 10.1111/ijd.14833. 32162331

[3] Al-Rawee R. Y. , Jawhar N. M. T. , and Saeed M. M. , Challenge Dilemma of Actinomycosis in the Tongue: Review and Case Report, International Journal of Surgery Case Reports. (2020) 75, 176–181, 10.1016/j.ijscr.2020.09.026. 32957074 PMC7505753

[4] Palonta F. , Preti G. , Vione N. , and Cavalot A. L. , Actinomycosis of the Masseter Muscle: Report of a Case and Review of the Literature, Journal of Craniofacial Surgery. (2003) 14, no. 6, 915–918, 10.1097/00001665-200311000-00015. 14600635

[5] Lan M. C. , Huang T. Y. , Lin T. Y. , and Lan M. Y. , Pathology quiz case 1. Actinomycosis of the Lip Mimicking Minor Salivary Gland Tumor, Archives of Otolaryngology–Head & Neck Surgery. (2007) 133, no. 4, 10.1001/archotol.133.4.411, 17438260. 17438260

[6] Matsuda S. , Yoshida H. , and Yoshimura H. , Orofacial Soft Tissues Actinomycosis: A Retrospective, 10-Year Single-Institution Experience, Journal of Dental Sciences. (2021) 16, no. 1, 365–369, 10.1016/j.jds.2020.01.003. 33384821 PMC7770242

[7] Bonnefond S. , Catroux M. , Melenotte C. , Karkowski L. , Rolland L. , Trouillier S. , and Raffray L. , Clinical Features of Actinomycosis: A Retrospective, Multicenter Study of 28 Cases of Miscellaneous Presentations, Medicine. (2016) 95, no. 24, e3923, 10.1097/MD.0000000000003923, 27311002. 27311002 PMC4998488

[8] Alamillos-Granados F. J. , Dean-Ferrer A. , García-López A. , and López-Rubio F. , Actinomycotic Ulcer of the Oral Mucosa: An Unusual Presentation of Oral Actinomycosis, British Journal of Oral and Maxillofacial Surgery. (2000) 38, no. 2, 121–123, 10.1054/bjom.1997.0373. 10864706

[9] Ateşpare A. , Keskin G. , Erçin C. , Keskin S. , and Camcıoğlu A. , Actinomycosis of the Tongue: A Diagnostic Dilemma, Journal of Laryngology & Otology. (2006) 120, no. 8, 681–683, 10.1017/S0022215106001757. 16716241

[10] Enoz M. , Actinomycosis of the Tongue, Journal of Infectious Diseases. (2006) 6, no. 1.

[11] Habibi A. , Salehinejad J. , Saghafi S. , Mellati E. , and Habibi M. , Actinomycosis of the Tongue, Archives of Iranian Medicine. (2008) 11, no. 5, 566–568. 18759530

[12] Kurtaran H. , Ugur K. S. , Ark N. , Vuran O. , and Gunduz M. , Tongue Abscess With Actinomycosis, Journal of Craniofacial Surgery. (2011) 22, no. 3, 1107–1109, 10.1097/SCS.0b013e3182108e9b. 21586957

[13] Escoda M. , Gardiello M. , and Muntané M. J. , Painful Tongue Ulcers, Actas Dermo-Sifiliográficas. (2013) 104, no. 1, 77–78, 10.1016/j.ad.2012.04.013. 22925228

[14] Rocha O. K. M. S. , de França G. M. , Oliveira P. T. , Santos J. L. M. , Miguel M. C. C. , and Silveira É. J. D. , Unusual Presentation of Oral Actinomycosis in the Tongue: Case Report, Jornal Brasileiro de Patologia e Medicina Laboratorial. (2017) 53, no. 5, 330–333, 10.5935/1676-2444.20170053.

[15] Jat P. S. , Paulose A. A. , and Agarwal S. , Lingual Actinomycosis, an Uncommon Diagnosis of Tongue Lesions: A Case Report and Review of Literature, Annals of Clinical Case Reports. (2017) 2.

[16] Aneja T. , Bhat N. , Chawla G. , and Negi M. , Actinomycosis of Tongue: Case Report of an Atypical Presentation, IOSR Journal of Dental and Medical Sciences. (2017) 16, no. 9, 84–86, 10.9790/0853-1609038486.

[17] Sadeghi S. , Azaïs M. , and Ghannoum J. , Actinomycosis Presenting as Macroglossia: Case Report and Review of Literature, Head and Neck Pathology. (2019) 13, no. 3, 327–330, 10.1007/s12105-018-0966-7. 30244331 PMC6684727

[18] Bilgen F. , Duman Y. , and Bekerecioglu M. , A Rare Location: Actinomyces in Tongue, Selcuk University Medical Journal. (2018) 34, no. 4, 180–182, 10.30733/std.2018.00995.

[19] Ahmed S. , Ali M. , Adegbite N. , Vaidhyanath R. , and Avery C. , Actinomycosis of Tongue: Rare Presentation Mimicking Malignancy With Literature Review and Imaging Features, Radiology Case Reports. (2019) 14, no. 2, 190–194, 10.1016/j.radcr.2018.10.022. 30425772 PMC6231289

[20] D′Amore F. , Franchini R. , Moneghini L. , Lombardi N. , Lodi G. , Sardella A. , and Varoni E. M. , Actinomycosis of the Tongue: A Case Report and Review of Literature, Antibiotics. (2020) 9, no. 3, 10.3390/antibiotics9030124, 32187989. PMC7148533 32187989

[21] Farquharson A. , Cotca C. C. , Helig A. , and Brown R. S. , Actinomycosis of the Ventral Tongue With Successful Laser Ablation Therapy: A Case Report, Oral Surgery, Oral Medicine, Oral Pathology and Oral Radiology. (2021) 132, no. 5, e175–e179, 10.1016/j.oooo.2021.07.017. 34489213

[22] Reyes M. R. T. , Dominguete M. H. L. , Javaroni J. B. , da Silveira H. A. , Silva E. V. , and León J. E. , Lingual Actinomycosis Clinically Simulating Nodular Median Rhomboid Glossitis: Literature Review and Report of Additional Case, Indian Journal of Otolaryngology and Head & Neck Surgery. (2023) 75, no. 4, 3984–3987, 10.1007/s12070-023-04045-0. 37974684 PMC10645888

[23] Bratzler D. W. , Dellinger E. P. , Olsen K. M. , Perl T. M. , Auwaerter P. G. , Bolon M. K. , Fish D. N. , Napolitano L. M. , Sawyer R. G. , Slain D. , Steinberg J. P. , Weinstein R. A. , American Society of Health-System Pharmacists , Infectious Disease Society of America , Surgical Infection Society , and Society for Healthcare Epidemiology of America , Clinical Practice Guidelines for Antimicrobial Prophylaxis in Surgery, American Journal of Health-System Pharmacy. (2013) 70, no. 3, 195–283, 10.2146/ajhp120568, 23327981. 23327981

[24] Kolm I. , Aceto L. , Hombach M. , Kamarshev J. , Hafner J. , and Urosevic-Maiwald M. , Cervicofacial Actinomycosis: A Long Forgotten Infectious Complication of Immunosuppression - Report of a Case and Review of the Literature, Dermatology Online Journal. (2014) 20, no. 5, 22640, 10.5070/D3205022640, 24852779. 24852779

[25] Vazquez A. M. , Marti C. , Renaga I. , and Salavert A. , Actinomycosis of the Tongue Associated With Human Immunodeficiency Virus Infection: Case Report, Journal of Oral and Maxillofacial Surgery. (1997) 55, no. 8, 879–881, 10.1016/S0278-2391(97)90354-2. 9251621

[26] Li Y. , Zhu L. , Yang W. , and You C. , Bloodstream Infection Caused by Coinfection of Actinomyces turicensis and Gemella morbillorum: A Case Report and Literature Review, Frontiers in Medicine. (2025) 12, 1626567, 10.3389/fmed.2025.1626567, 40861233. 40861233 PMC12370484

[27] Khan Z. R. , Hanbali L. , Majeed I. , Wadhwa S. , Mehta A. , and Arnold F. W. , Actinomyces in the Bloodstream: A Pathogen or Passenger?, Cureus. (2025) 17, no. 7, e88001, 10.7759/cureus.88001. 40821199 PMC12352521

[28] Frickmann H. , Theis F. , and Warnke P. , Relevance in Question: A Rare Case of Actinomyces radicidentis Bacteremia, European Journal of Microbiology and Immunology. (2025) 15, no. 3, 157–163, 10.1556/1886.2025.00048. 40952804 PMC12505146

